# Government Getting Us Good Groceries

**Published:** 1890-07

**Authors:** S. H. Preston


### GOVERNMENT GETTING US GOOD GROCERIES.

BY S. H. PRESTON.

The Senate Committee on Agriculture and Forestry, in Washington, through Mr. Paddock, has submitted a substitute for the Pure Food bill introduced in the Senate. The substitute provides for the creation of a food division of the Agricultural Department, with a corps of chemists, inspectors, etc., and makes it a misdemeanor for any person to knowingly manufacture in the United States, or ship from any foreign country to the United States, an article of food or any drug which is adulterated so as to be injurious to health. The substitute bill is practically the same as the Senate bill.

Nine years ago, a bill introduced by Senator Anderson, of Wisconsin, imposing penalties for neglect to label adulterated commodities as such, awakened a sort of amateur agitation on this subject, and elicited some startling reports from chemical experts respecting the extensive use of adulterants in the grocery trade. One of these stated that out of fourteen specimens of sugar, analysis showed twelve of them to be adulterated, not only with glucose, but with chloride of tin. Glucose is bad enough, especially for the kidneys, but chloride of tin is an active poison. Another chemist discovered organic substances and various fungi in oleomargarine, which he ascribed to the use of the stomachs of pigs and sheep, in the preparation of this modern substitute for butter. It was found that the common qualities of tin used in the canning of meats, fruits, and vegetables, imparted poisonous properties to their contents. The laboratories of these chemists showed the following :

Bread, adulterated with alum and sulphate of copper ; yeast, with alum ; baking powder, with alum, terra alba, plaster of paris, whiting, and kaolin ; milk, with a variety of articles ; cheese, with potatoes, beans, oleomargarine, vermillion, red chalk, sulphate of copper, arsenic, and corrosive sublimate ; lard, with boiled starch, alum, and quicklime; confectionery, with chromote of lead, red lead, vermillion, Prussian blue, copper, and arsenic ; pickles, with sulphuric acid and verdigris ; mustard, with yellow ochre and chromate of lead ; vinegar, with sulphuric acid, arsenic, and corrosive sublimate ; coffee, with roasted acarno, spent tan bark, logwood, mahogany, sawdust, and burnt liver of horses ; teas, with a great variety of ingredients.

After such a scientific showing it is not surprising that nervous and dyspeptic statesmen should give the subject some attention. It is unquestionably within the province of the general government to inspect and regulate any branch of commerce which imperils health and life. The passage and enforcement of the Paddock Pure Food bill will present a new chapter in our criminal jurisprudence. The traffic in such adulterated articles of diet is simply a system of wholesale assassination, and the murderers of the markets should be punished with the severity of other evil doers.

